# Motility defects in *Campylobacter jejuni* defined gene deletion mutants caused by second-site mutations

**DOI:** 10.1099/mic.0.000184

**Published:** 2015-12

**Authors:** Stefan P. W. de Vries, Srishti Gupta, Abiyad Baig, Joanna L'Heureux, Elsa Pont, Dominika P. Wolanska, Duncan J. Maskell, Andrew J. Grant

**Affiliations:** Department of Veterinary Medicine, University of Cambridge, Cambridge, UK

## Abstract

Genetic variation due to mutation and phase variation has a considerable impact on the commensal and pathogenic behaviours of *Campylobacter jejuni*. In this study, we provide an example of how second-site mutations can interfere with gene function analysis in *C. jejuni*. Deletion of the flagellin B gene (*flaB*) in *C. jejuni* M1 resulted in mutant clones with inconsistent motility phenotypes. From the *flaB* mutant clones picked for further analysis, two were motile, one showed intermediate motility and two displayed severely attenuated motility. To determine the molecular basis of this differential motility, a genome resequencing approach was used. Second-site mutations were identified in the severely attenuated and intermediate motility *flaB* mutant clones: a TA-dinucleotide deletion in *fliW* and an A deletion in *flgD*, respectively. Restoration of WT *fliW*, using a newly developed genetic complementation system, confirmed that the second-site *fliW* mutation caused the motility defect as opposed to the primary deletion of *flaB*. This study highlights the importance of (i) screening multiple defined gene deletion mutant clones, (ii) genetic complementation of the gene deletion and ideally (iii) screening for second-site mutations that might interfere with the pathways/mechanisms under study.

## Introduction

*Campylobacter jejuni* is the leading bacterial cause of foodborne gastroenteritis worldwide. The impact of *C. jejuni* infections is significant due to their high incidence, duration of infection and possible post-infection sequelae (Ruiz-Palacios, 2007). *C. jejuni* has a broad range of environmental reservoirs including water, birds and other domestic animals, with chickens representing the largest source of human infection (Young *et al.*, 2007). Although *C. jejuni* is considered as commensal in chickens, it has a significant impact on animal welfare in certain breeds of bird (Humphrey *et al.*, 2014). The genome of *C. jejuni* is subject to considerable genetic variation, which is thought to play an important role in the survival of the species within host organisms and the environment, especially by altering surface exposed structures such as flagella, lipooligosaccharide and capsular polysaccharide (Balaban *et al.*, 2009; Jerome *et al.*, 2011; Joslin & Hendrixson, 2009; Mohawk *et al.*, 2014; Thomas *et al.*, 2014). Genetic heterogeneity arises in the *C. jejuni* population as a consequence of mutations due to replication errors, phase variation of homopolymeric or repetitive heteropolymeric tracts either in coding sequences or in promoter regions and to a lesser extent, recombination (Thomas *et al.*, 2014; Wassenaar, 2011; Wilson *et al.*, 2009). It has been suggested that the lack of a functional mismatch repair system enhances the frequency of phase variation and random mutations, and thus the overall heterogeneity of the *C. jejuni* population (Gaasbeek *et al.*, 2009).

Flagellar-mediated motility plays a central role in commensal and pathogenic behaviours of *C. jejuni* (Guerry, 2007; Lertsethtakarn *et al.*, 2011). The flagellar system is subject to genetic variation at multiple levels, which often leads to alteration of motility (Gao *et al.*, 2014; Guerry, 2007; Hendrixson & DiRita, 2004). *C. jejuni* possesses polar flagella at one or both ends of the bacterial cell which consist of a membrane-embedded basal body, a hook structure and a protruding polymeric filament that is composed of the major flagellin subunit FlaA and the minor subunit FlaB (Gao *et al.*, 2014; Guerry *et al.*, 1991). In *C. jejuni* strain 81116, inactivation of *flaA* led to a non-flagellated and non-motile phenotype, whereas flagella and motility were retained after the inactivation of *flaB* (Nuijten *et al.*, 1990; Wassenaar *et al.*, 1991). The motor subunits at the flagellar base, encoded by *motA* and *motB*, are responsible for flagellar rotation. Mohawk *et al.* (2014) reported that non-motile variants of *C. jejuni* 81-176 carried distinct mutations [i.e. single nucleotide polymorphisms (SNPs) and insertions and deletions (INDELs)] in the *motA* gene. Flagellar gene expression is tightly controlled and requires the alternative δ^54^ and δ^28^ factors, the FlgSR two-component system, the FlhF GTPase and the flagellar secretion system (Balaban *et al.*, 2009; Hendrixson & DiRita, 2003; Joslin & Hendrixson, 2009). Phase variation mechanisms acting on the FlgSR system that affect motility in *C. jejuni* 81-176 include reversible phase variation in (i) homopolymeric poly-A and poly-T tracts within the *flgR* response regulator (Hendrixson, 2006) and (ii) poly-A tracts and heteropolymeric repeats located in the *flgS* sensor histidine kinase (Hendrixson, 2008). It is noteworthy that the *C. jejuni* flagellar filament is extensively glycosylated, predominantly with pseudaminic acid and legionaminic acid and derivatives thereof. Recombination within the flagellar glycosylation gene locus and homopolymeric poly-G tract phase variation are mechanisms of variation (Howard *et al.*, 2009; Karlyshev *et al.*, 2002; van Alphen *et al.*, 2008).

Here we show that the propensity for stochastic mutation events in *C. jejuni* has clear implications for gene function analysis in an experimental setting. Analysis of *C. jejuni* M1 *flaB* mutant clones revealed inconsistent motility phenotypes. Using genome resequencing and in-depth genotypic and phenotypic characterization, we identified second-site mutations and confirmed that these were responsible for the observed inconsistent motility amongst *flaB* mutant clones. We also describe a new versatile and user-friendly system for genetic complementation in *C. jejuni*.

## Methods

### Bacterial strains and growth conditions

All WT strains, defined mutants, genetically complemented mutants and plasmids are summarized in Table 1. *C. jejuni* strain M1 was originally isolated from a researcher after visiting a poultry processing plant (Friis *et al.*, 2010) and is fully motile and spiral-shaped. *C. jejuni* M1 was routinely cultured on brain heart infusion (BHI, Oxoid) agar plates supplemented with 5 % defibrinated horse blood (Oxoid) and 5 μg trimethoprim ml^− 1^ (TrM). Gene deletion mutants were cultured in the presence of 10 μg chloramphenicol ml^− 1^ and genetically complemented mutant strains were also supplemented with 50 μg kanamycin ml^− 1^. *C. jejuni* was cultured under microaerophilic conditions (5 % O_2_, 10 % CO_2_ and 85 % N_2_) in a MACS VA500 Variable Atmosphere Work Station (Don Whitley). *Escherichia coli* NEB 5α or 10-β (New England Biolabs) was used for cloning and was cultured in LB medium at 37 °C, supplemented as appropriate with antibiotics.

**Table 1.** Bacterial strains and plasmids used in this study

Strain or plasmid *	Relevant genotype† or description	Source/reference
***C. jejuni***		
M1	WT	Friis *et al.* (2010)
M1 coupled to Δ*flaB*	WT	This study
M1 coupled to ΔflaB*, Δ*flaA,* Δ*flaAB*	WT	This study
M1 coupled to Δ*flaB*+empty GC	WT; Km^r^	This study
M1 coupled to Δ*flaD,* Δ*pflA*	WT	This study
M1 coupled to Δ*fliW*	WT	This study
M1 coupled to Δ*fliW*+empty GC	WT; Km^r^	This study
M1 Δ*flaB* clones A, B, C, D, E	ΔCJM1_1294; Cm^r^	This study
M1 ΔflaB* clones A, B, C	ΔCJM1_1295-1294; Cm^r^	This study
M1 Δ*flaA* clones A, B, C	ΔCJM1_1296; Cm^r^	This study
M1 Δ*flaAB* clones A, B, C	ΔCJM1_1295-1294/CJM1_1296; Cm^r^	This study
M1 Δ*flaD* clones A, B, C	ΔCJM1_0851; Cm^r^	This study
M1 Δ*pflA* clones A, B, C	ΔCJM1_1501; Cm^r^	This study
M1 Δ*fliW* clone A, B, C	ΔCJM1_1052; Cm^r^	This study
M1 Δ*fliW* clone A+empty GC	ΔCJM1_1052; Cm^r^ Km^r^	This study
M1 Δ*fliW* clone A+*fliW* GC	ΔCJM1_1052+CJM1_1052; Cm^r^ Km^r^	This study
M1 Δ*flaB* clone D+empty GC	ΔCJM1_1294; Cm^r^ Km^r^	This study
M1 Δ*flaB* clone D+*fliW* GC	ΔCJM1_1294+CJM1_1052; Cm^r^ Km^r^	This study
M1 Δ*flaB* clone E+empty GC	ΔCJM1_1294; Cm^r^ Km^r^	This study
M1 Δ*flaB* clone E+*fliW* GC	ΔCJM1_1294+CJM1_1052; Cm^r^ Km^r^	This study
***E. coli* strains**		
NEB 10-β or 5-α	Host strains for pSV009 and derivatives thereof	New England Biolabs
**Plasmids**		
pCC027	Cm^r^ donor plasmid	Coward *et al.* (2008)
pMiniT	*E. coli* PCR cloning plasmid	New England Biolabs
pUC19	*E. coli* cloning plasmid	New England Biolabs; Yanisch-Perron *et al.* (1985)
pSV009	*C. jejuni g*enetic complementation plasmid, pUC19 backbone; donor for empty GC region	This study
pSV012	pSV009 containing *fliW*; donor for *fliW* GC region	This study

* empty GC, Empty genetic complementation region amplified from pSV009; *fliW* GC, genetic complementation region containing the *fliW* promoter and coding sequence amplified from pSV012.

† Cm^r^, chloramphenicol resistance; Km^r^, kanamycin resistance.

### Electroporation and natural transformation of *C. jejuni*

Electroporation of *C. jejuni* was performed as described previously (Holt *et al.*, 2012) with minor adjustments. *C. jejuni* M1 was cultured on BHI-TrM blood agar plates for ∼48 h then replated onto fresh BHI-TrM blood agar plates and grown for ∼16 h. Bacteria were harvested from plates in BHI broth, washed four times in ice-cold wash buffer [272 mM sucrose and 15 % (v/v) glycerol] and then resuspended in wash buffer. Of this suspension, 100 μl was added per 0.2 cm pre-chilled electroporation MicroPulser cuvette (Bio-Rad) and mixed with DNA (∼3 μg). Electroporation was performed using a GenePulser Xcell system (Bio-Rad) using the following settings: 2.5 kV, 200 Ω and 25 μF. After a single pulse, 200 μl of pre-warmed SOC medium was added and the suspension was transferred onto 2 ml BHI-TrM blood agar in a Universal tube (Greiner). After 5 h incubation under microaerophilic conditions, 1 ml of pre-warmed BHI medium was added to resuspend the bacteria, which were plated onto selective agar plates and grown under microaerophilic conditions for 2 to 3 days.

Natural transformation of *C. jejuni* was performed using an adapted biphasic method (Holt *et al.*, 2012; van Vliet *et al.*, 1998). *C. jejuni* was grown on BHI-TrM blood agar plates for ∼48 h and replated onto fresh BHI-TrM blood agar plates and grown for ∼16 h. Bacteria were harvested from plates in BHI broth and the OD_600_ was adjusted to ∼0.5. Of this suspension, 0.5 ml was pipetted onto 2 ml BHI-TrM blood agar in a Universal tube (Greiner) and incubated for 3 h under microaerophilic conditions. Thereafter, DNA was added and incubated for 3–5 h before bacteria were harvested and then plated on selective agar plates and grown for 2 to 3 days under microaerophilic conditions.

### Construction of defined gene deletion mutants

*C. jejuni* defined gene deletion mutants were generated by allelic replacement of the gene with a chloramphenicol resistance cassette (*cat*). The *cat* cassette was amplified by PCR from the plasmid pCC027 (Coward *et al.*, 2008) and the 5′ (L1 and L2 primers) and 3′ (R1 and R2 primers) flanking regions of the target gene were amplified by PCR from *C. jejuni* M1 genomic DNA. PCRs were performed using Q5 DNA polymerase (New England Biolabs) and purified using the QIAquick PCR purification kit (Qiagen). The gene flanking region PCR primers L2 (reverse primer 5′ flank) and R1 (forward primer 3′ flank) contain extensions that are complementary to the primers used for amplification of the *cat* cassette. The *cat* cassette (640 ng) and gene flanking regions (200 ng each) were joined in an overlap PCR without primers, resulting in the incorporation of the *cat* cassette in between the 5′ and 3′ flanking regions. Two microlitres of the product of this overlap PCR were then used as a template in a second round of amplification, this time using the primers L1 (forward primer 5′ flank) and R2 (reverse primer 3′ flank), which flank the original gene sequence and thus amplify the whole fragment; a total of four 50 μl PCRs were performed for each mutant, and subsequently pooled and purified. Of this, ∼3 μg of the overlap PCR product was used for electroporation of *C. jejuni* M1 to generate first generation defined gene deletion mutants, which were selected on BHI-TrM-Cm blood agar. Next, first generation mutant DNA was isolated using the DNeasy Blood and Tissue kit (Qiagen) and checked by PCR for recombination at the desired genomic location using the L1 forward primer (5′ gene flanking region) and a control reverse primer located in the gene (‘A’ primer, which should not generate a product) and a *cat* cassette primer (CC069, which should generate a product of predicted size). First generation gene deletion mutant DNA (50 ng) was used for natural transformation of *C. jejuni* M1 WT; the gene replacement event was selected and confirmed by PCR. For *flaB* deletion mutant clones, correct integration of the *cat* cassette was also analysed by DNA sequencing. In addition, the WT was processed in parallel through the transformation procedure without any added mutagenic DNA to obtain a ‘coupled’ WT strain. This was done to reduce the genetic variation between the WT and defined mutant strains. Primer sequences are given in Table S1.

### Motility assay

*C. jejuni* was grown on BHI blood agar plates for ∼48 h then replated onto fresh BHI blood agar plates and grown for ∼16 h. BHI suspensions of OD_600_ ∼0.5 were made and stabbed, using a 20 μl pipette tip, into the middle of a 9 cm petri dish containing 25 ml of 0.4 % select agar (Sigma Aldrich) BHI medium and grown overnight under microaerophilic conditions. The diameter of the ring of bacterial growth was measured using a ruler (*n* = 3).

### Genetic complementation

For genetic complementation of defined gene deletion mutants, the target gene was inserted into a pseudogene region between CJM1_0055 and CJM1_0057 (Coward *et al.*, 2008). For this, a new genetic complementation system was designed *in silico* and synthesized *de novo* from oligonucleotides (GeneArt Strings, Life Technologies). The genetic complementation system consists of the *cat* promoter region from pRY011 (Coward *et al.*, 2008; Yao *et al.*, 1993), a partial pUC19 multiple cloning site and the kanamycin resistance cassette including its promoter region from pRY107 (Yao *et al.*, 1993), all of which is flanked by 400 bp of 5′ and 3′ sequence of the CJM1_0055–0057 pseudogene region (98–100 % identity with sequences in *C. jejuni* 11168, 81116 and 81-176), see Fig. 5(a) for a schematic overview. The *de novo* synthesized genetic complementation region was first cloned into pMiniT using the NEB PCR cloning kit (New England Biolabs) and subsequently subcloned into pUC19 using the *Hin*dIII and *Eco*RI restriction enzyme sites, yielding the plasmid pSV009. Of note, the 5′ pseudogene region (CJM1_0055–0057) sequenced originally harboured a *Hin*dIII site, which was eliminated by introducing a synonymous mutation during the *in silico* design of the genetic complementation insert. The *fliW* coding sequence including its upstream sequence was amplified by PCR and subcloned into pSV009 using the *Xho*I and *Bam*HI sites, yielding pSV012-*fliW*. Sequences of pSV009 and pSV012-*fliW* were confirmed using DNA sequencing. The *fliW* complementation region and the empty genetic complementation region were amplified by PCR from pSV012 or pSV009, respectively, using the primers pSV009_GCampl_FW1/RV1, which amplify the complete genetic complementation region. The *fliW* complementation or the empty genetic complementation region was subsequently introduced into *C. jejuni* by electroporation. For a schematic overview of the genetic complementation strategy, see Fig. 5(a). Primer sequences are given in Table S1.

### Scanning and transmission electron microscopy (EM) analysis

*C. jejuni* M1 WT and defined gene deletion mutants were cultured for ∼48 h, harvested and washed four times in water. For scanning EM, bacteria were fixed in 2 % glutaraldehyde in 0.05 M sodium cacodylate buffer at pH 7.4 for 48 h, allowed to settle onto 10 mm poly-l-lysine-coated glass coverslips, rinsed twice in deionized water and quench frozen in propane cooled in liquid nitrogen. Samples were freeze-dried overnight from − 95 to +30 °C in a K775X freeze dryer (Quorum Emitech). The coverslips were mounted on Cambridge stubs with silver DAG and coated with 4 nm of gold in a K575X sputter coater (Quorum Emitech), then imaged using a Verios 460 (FEI) scanning electron microscope operated at 2 or 5 kV. For transmission EM, bacteria were fixed in 2 % glutaraldehyde in 0.05 M sodium cacodylate buffer at pH 7.4 for 6–12 h, and adsorbed onto glow discharged 400 mesh copper grids with a carbon film attached for 30 s. They were rinsed twice with deionized water and stained for 30 s with 1 % aqueous uranyl acetate. Grids were allowed to dry for 20 min and viewed in a Tecnai G2 (FEI) transmission electron microscope operated at 200 keV. Images were captured with an AMT XR60B camera running Deben software.

### Illumina sequencing

Genomic DNA was isolated using Genomic-tip 20/G columns (Qiagen) or the DNeasy Blood and Tissue kit (Qiagen) according to manufacturer's instructions. Libraries for Illumina sequencing were prepared using the NEBNext Ultra DNA library prep kit (New England Biolabs) or the Nextera XT DNA kit (Illumina). For the NEBNext Ultra DNA kit, the DNA was first sheared to ∼300 or 400 bp fragments in microTUBE screw-cap tubes in an M220 focused-ultrasonicator (Covaris). Following NEBNext Ultra DNA library preparation, the library size was determined with a Bioanalyser 2100 (Agilent) and the DNA concentration was measured with the Qubit dsDNA BR kit (Life Technologies) and pooled at equimolar amounts. Nextera XT library preparation was performed according to the manufacturer's instructions. The libraries for *pflA* (CJM1_1501), *flaD* (CJM1_0851), *flaB* (CJM1_1294) gene deletion mutant clones and their coupled WT strains were sequenced using 76 bp paired-end sequencing, whereas the *fliW* gene deletion mutant clone A and its coupled WT were sequenced on the Illumina MiSeq platform using 301 bp paired-end sequencing (v3 chemistry).

### Illumina sequence data analysis

Raw sequence reads for *pflA* (CJM1_1501), *flaD* (CJM1_0851), *flaB* (CJM1_1294) mutant clones and their coupled WTs were mapped to the M1 reference genome sequence [GenBank accession no. CP001900 (Friis *et al.*, 2010)] using the CLC Genomics Workbench version 7.5.1. Variants were called only if covered by >20 reads, a variant count of >5 reads and detected in both forward and reverse sequence reads. Hierarchical clustering based on SNPs and INDELs presence/absence was performed using CLC Main Workbench (v7); variants present in all sequenced mutants and WTs were excluded from the clustering analysis.

Variant analysis of *fliW* mutant clone A and its coupled WT was performed as follows: (1) Illumina sequence reads were mapped to the M1cam reference genome (accession no. CP012149, see details below) using Stampy (Lunter & Goodson, 2011), (2) Samtools was used to identify variants (Li *et al.*, 2009); the minimum quality for variant calling was set to 100. The variant effect at the protein level was predicted using SnpEff (Cingolani *et al.*, 2012).

### Genome resequencing and annotation of *C. jejuni* M1 ‘Cambridge’

The M1 WT strain used in our laboratory and ‘coupled’ WT strains (i.e. the M1 WT processed through the natural transformation procedure) obtained during the generation of *flaB* (one WT), *pflA* and *flaD* (two WTs) gene deletion mutants were Illumina sequenced and their sequence reads mapped to the M1 reference sequence [GenBank accession no. CP001900 (Friis *et al.*, 2010)]. SNPs and INDELs detected in all four sequenced M1 WTs were collected and incorporated into the *C. jejuni* M1 reference genome sequence using a custom Python script, yielding an in-house *C. jejuni* M1 genome sequence (designated CJM1cam). The effect of SNPs and INDELs at the protein level was predicted using SnpEff (Cingolani *et al.*, 2012). Sequence variants detected in this study by Sanger DNA sequencing were also incorporated into the CJM1cam genome sequence. An overview of the SNPs and INDELs relative to the published M1 reference genome can be found in Table S2. The obtained CJM1cam genome sequence was annotated with Prokka (Seemann, 2014) and assigned locus tags starting with CJM1cam; where possible the locus tag numbering of the original M1 reference genome was retained. The annotation of the flagellin locus CJM1_1295-1294 (*flaB*) and CJM1_1296 (*flaA*) was manually corrected. Automated ORF calling using Prokka resulted in the convergence of 33 partial coding sequences (CDSs) into 16 CDSs relative to the published M1 genome (Friis *et al.*, 2010). The CJM1cam proteins were assigned Cluster of Orthologous Genes (Tatusov *et al.*, 1997) functional categories using a custom R script. The obtained CJM1cam genome sequence and annotation are available through GenBank (accession no. CP012149).

### Statistical analyses

Statistical analyses were performed in GraphPad Prism 6.0 (GraphPad software) with *P* < 0.05 considered to be significant.

### Sequencing data

The CJM1cam genome sequence is deposited at GenBank (CP012149) and the sequence of the pSV009 genetic complementation plasmid is deposited KT373982. The Illumina sequence data are deposited at the European Nucleotide Archive under study accession number PRJEB10223 (http://www.ebi.ac.uk/ena).

## Results and discussion

### Differential motility of defined gene deletion mutant clones

**Fig. 1.**
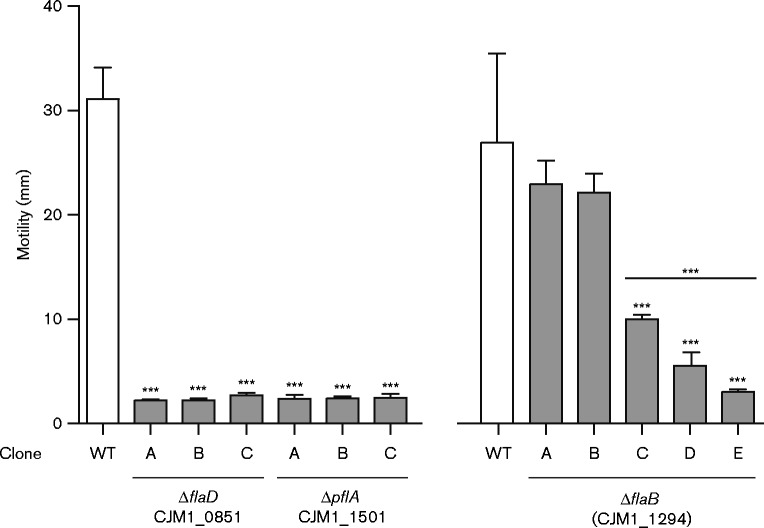
Motility of *C. jejuni* M1 flagella defined gene deletion mutants. Motility was analysed on BHI plates with 0.4 % (w/v) agar after overnight incubation. Three *flaD* (CJM1_0851) and *plfA* (CJM1_1501) deletion mutant clones were assayed along with their coupled WT strain and five *flaB* [CJM1_1294, previously annotated to encode FlaA (Friis *et al.*, 2010)] mutant clones were tested with two coupled WT strains (A and B), with the average motility shown for the latter. All three *flaD* (CJM1_0851) and *pflA* (CJM1_1501) mutant clones were non-motile; however, motility of *flaB* (CJM1_1294) mutant clones was inconsistent: clones A and B showed WT motility, clone C displayed intermediate motility, whereas the motility of clones D and E was severely attenuated. Differential motility of mutant clones was tested against the coupled WT strain(s) with a Mann–Whitney test (*n* = 3). Data shown are the mean and sem, with ***P < 0.001.

For our studies into the molecular mechanisms of *C. jejuni* pathogenesis, e.g. invasion of gut epithelial cells (to be reported elsewhere), three defined deletion mutants were generated in *C. jejuni* M1 flagella genes using an overlap PCR method. Gene deletions were constructed in *flaD*, also referred to as *flgL* (CJM1_0851; hook filament junction protein) (Neal-McKinney & Konkel, 2012), *pflA* (CJM1_1501; paralysed flagellum protein) (Yao *et al.*, 1994) and CJM1_1294, predicted to encode the major flagellin subunit FlaA (CJM1_1294) in the published *C. jejuni* M1 reference genome (Friis *et al.*, 2010). We anticipated that deletion of these flagella genes would result in non-motile phenotypes (Neal-McKinney & Konkel, 2012; Wassenaar *et al.*, 1991; Yao *et al.*, 1994). As expected (Neal-McKinney & Konkel, 2012; Yao *et al.*, 1994) three independent mutant clones for each of *flaD* (CJM1_0851) and *pflA* (CJM1_1501) were found to be non-motile (Fig. 1). Scanning EM confirmed the previously reported non-flagellated and flagellated phenotype of *flaD* and *pflA* gene deletion mutants, respectively (data not shown) (Neal-McKinney & Konkel, 2012; Yao *et al.*, 1994). In contrast, five independent *flaA* (CJM1_1294) mutant clones were tested for motility with inconsistent results: mutant clones A and B displayed WT motility, clone C showed intermediate motility and clones D and E had severely attenuated motility (Fig. 1).

**Fig. 2.**
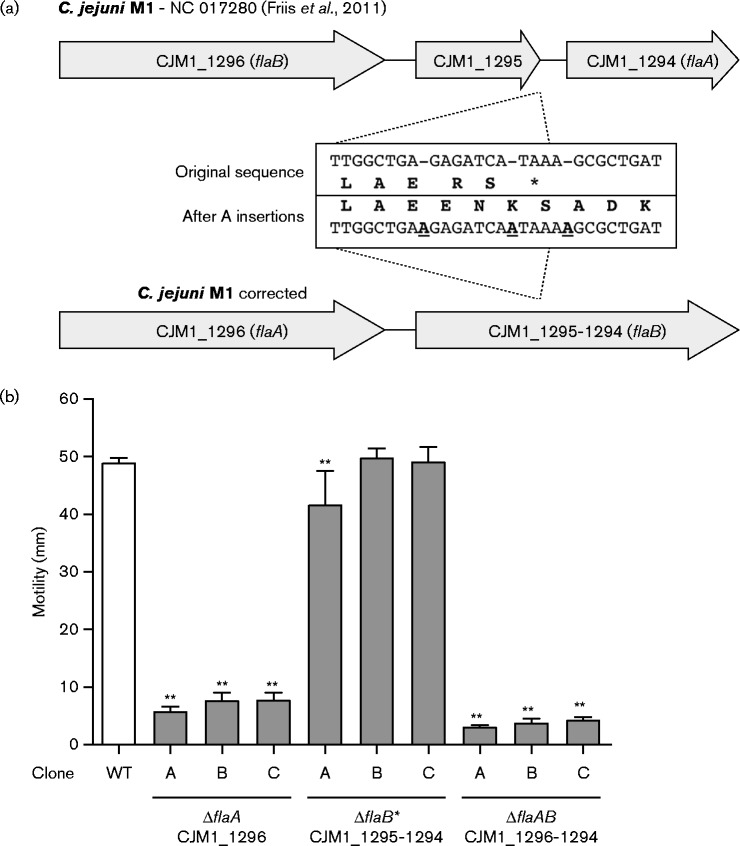
Sequence corrections in the *C. jejuni* M1 flagellin locus and motility analysis of flagellin locus gene deletion mutants. (a) Insertion of three A nucleotides at the 3′ of CJM1_1295 led to elimination of the TAA stop codon and resulted in CJM1_1295 and CJM1_1294 being merged into one large ORF. (b) Motility was assessed on 0.4 % (w/v) agar BHI plates after overnight incubation. Deletion of the full *flaB* CDS (flaB* mutant; CJM1_1295–1294) did not affect motility in clones B and C; however, we observed a very marginal decrease in motility in clone A. In contrast, mutant clones in which *flaA* (CJM1_1296) or both the *flaA* and *flaB* gene (*flaAB*; CJM1_1296 and CJM1_1295–1294) were deleted all showed severely attenuated motility. Differential motility of mutant clones was tested against the coupled WT strain with a Mann–Whitney test (*n* = 3). Data shown are the mean and sem, with **P < 0.01.

To unravel the basis of the inconsistent motility phenotypes of *flaA* (CJM1_1294) mutant clones, we first confirmed the correct insertion of the chloramphenicol (*cat*) resistance cassette in *flaA* (CJM1_1294) by Sanger sequencing. Importantly, this revealed the insertion of three A nucleotides in an 11 bp stretch at the 3′ end of CJM1_1295 [annotated as ‘hypothetical protein’ (Friis *et al.*, 2010)], which was also independently confirmed by Sanger sequencing of the complete CJM1_1295–1294 region. Incorporation of the A insertions into the original M1 sequence (Friis *et al.*, 2010) led to CJM1_1295 and CJM1_1294 being merged into a larger ORF of 1731 bp (Fig. 2a). The difference between the published *C. jejuni* M1 reference genome (Friis *et al.*, 2010) and the sequence of our M1 strain may reflect genotypic variation or sequencing or assembly errors in the original M1 genome sequence.

In-depth analysis of the altered flagellin locus in *C. jejuni* M1 revealed that: (1) the merged CJM1_1295–1294 gene actually represents the full-length *flaB* gene as it was identical to the published *flaB* sequence of *C. jejuni* 81116 (Nuijten *et al.*, 1990; Pearson *et al.*, 2007) and (2) CJM1_1296 was incorrectly annotated as *flaB*; it is identical to *flaA* of *C. jejuni* 81116 (Friis *et al.*, 2010), see Fig. 2(a). Therefore, our CJM_1294 gene deletion mutant, described above, is not a *flaA* mutant but the *cat* cassette is replacing a region ranging from position 822 to 1671 bp of the *flaB* gene. Consequently, the motile phenotype of *flaB* (CJM1_1294) mutant clones (Fig. 1) confirmed the findings by Wassenaar *et al.*, (1991), reporting a motile phenotype after the inactivation of *flaB* in *C. jejuni* 81116. Deletion of the full-length *flaB* gene (CJM1_1295–1294, referred to as the flaB* mutant) also resulted in WT motility levels for mutant clones B and C, although the motility of flaB* (CJM1_1295–1294) mutant clone A was slightly lower compared to the WT (Fig. 2b). In line with previous findings of Wassenaar *et al.* (1991) in *C. jejuni* 81116, deletion of *flaA* (CJM1_1296) and the complete flagellin locus (*flaAB*; CJM1_1296 and CJM1_1295–1294) led to abolished motility (Fig. 2b). Analysis of the combined motility data for the *flaA* (CJM1_1296) and *flaAB* mutant clones showed that deletion of the complete flagellin locus resulted in more severely attenuated motility compared to deletion of *flaA* alone (*P* < 0.001), suggesting that *flaB* plays a minor role in motility, at least in *C. jejuni* strain M1.

Based on our findings, we hypothesized that the attenuated motility in *flaB* (CJM1_1294) mutant clones C, D and E might be caused by loss-of-function mutations in other motility-associated genes or as previously reported, by a compensatory mechanism, for example, by increased *flaB* expression or by genome rearrangements, which have been shown to (partially) restore motility (Nuijten *et al.*, 2000; Wassenaar *et al.*, 1994, 1995).

### Genotypic and phenotypic analysis of motility defects

**Fig. 3.**
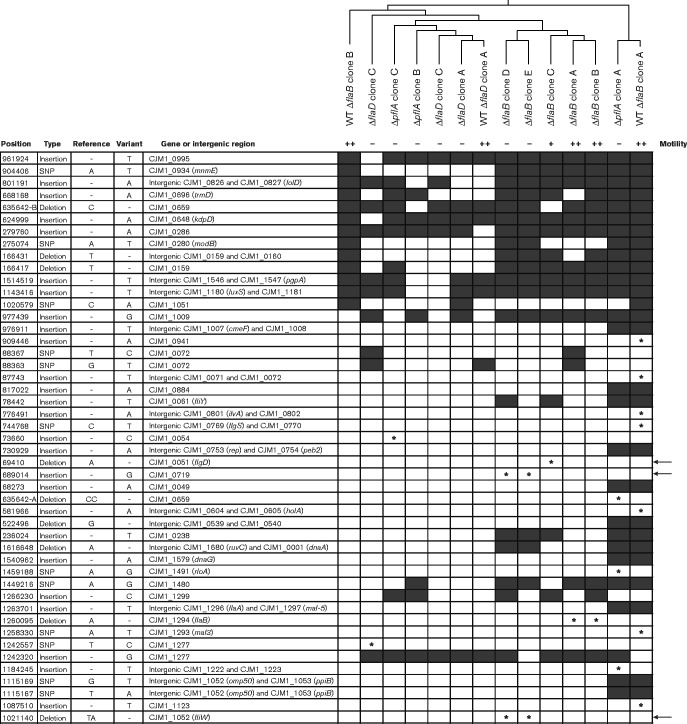
Genome sequencing revealed second-site mutations associated with motility defects. Genetic variation, i.e. SNPs and INDELs, in *C. jejuni* M1 defined gene deletion mutant clones and corresponding WT strains (Table 1) were analysed by Illumina sequencing; their presence is indicated with black boxes or with an asterisk when either found to be unique to individual mutant clones, WT strains, or associated with differential motility. Motility phenotypes are indicated as severely attenuated motility ( − ), intermediate motility (+) or WT motility (++) (see Fig. 1). SNPs or INDELs that are associated with attenuated motility in *flaB* (CJM1_1294) mutant clones C, D and E are indicated with an arrow on the right side of the panel. SNPs/INDELs that were detected in all sequenced M1 WTs and mutant strains compared to the M1 reference (Friis *et al.*, 2010) were excluded from the analysis (Table S2). Hierarchical clustering was performed based on the presence/absence of SNPs and INDELs.

**Fig. 4.**
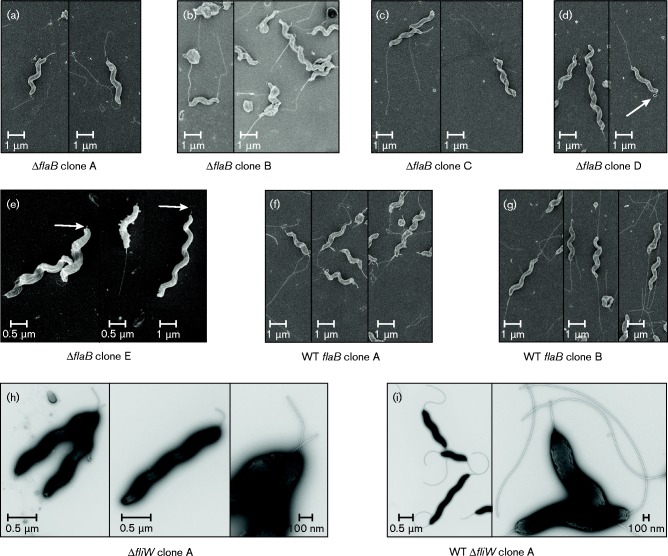
EM analysis of the flagellar structure in *flaB* (CJM1_1294) and *fliW* (CJM1_1052) gene deletion mutants. Scanning EM was used to assess the flagellar structure in *flaB* (CJM1_1294) mutant clones A–E (a–e) and coupled WT clones A and B (f, g) and transmission EM was used for analysis of the *fliW* deletion mutant clone A (h) and its parental WT (i). (a–c) The motile (clones A and B) and intermediate motility (clone C) *flaB* (CJM1_1294) mutants harboured bipolar flagella. (d, e) Non-motile *flaB* clones D and E lack flagellar structures at both poles; a protruding structure (flagellar filament or hook-like structure) was observed (indicated with white arrows), or rarely bacteria were observed that harboured a flagellar structure at a single pole as shown in the right panels of (d) and (e). (f, g) Motile WT isolates (clones A and B) coupled to *flaB* mutants displayed flagellar structures at both poles. (h) No flagellar filaments were observed for the non-motile *fliW* deletion mutant clone A; however, short hook-like structures were observed. (i) The WT isolate coupled to the *fliW* mutant clone A possessed flagellar structures at both poles.

A genome resequencing approach was used to assess whether second-site mutations might be responsible for the attenuated motility of *flaB* (CJM1_1294) mutant clones C, D and E. Three *pflA*, three *flaD* and five *flaB* mutant clones and their ‘coupled’ M1 WT strains were genome sequenced. Eighty-one variants, i.e. SNPs and INDELs, were detected relative to the *C. jejuni* M1 reference genome [GenBank CP001900 (Friis *et al.*, 2010)]. Of these, 34 SNPs and INDELs were present in all sequenced gene deletion mutant clones and their ‘coupled’ WTs, whereas 47 SNPs and INDELs were only detected in a subselection of them (Fig. 3). Although the *flaB* mutant clones subclustered, limited mutant-specific hierarchical clustering was detected, indicating that variation may be introduced throughout all growth steps during the construction of the gene deletion mutants. The SNPs and INDELs nucleotide position numbers correspond to the position in the original *C. jejuni* M1 reference genome sequence (Friis *et al.*, 2010). Interestingly, the genome resequencing approach revealed the presence of SNPs and INDELs unique to the severely attenuated motility *flaB* (CJM1_1294) mutant clones D and E. These were an insertion of a G at position 689 014 within CJM1_0719 (encoding a putative periplasmic protein) and deletion of a TA-dinucleotide at position 1 021 140 within CJM1_1052 (*fliW*). The TA-deletion in *fliW* is predicted to result in a three amino acid C-terminal truncation of the FliW protein. FliW encodes a predicted flagellin filament-stabilizing chaperone, although it has not yet been demonstrated whether FliW interacts with FlaA and/or FlaB flagellin subunits (Barrero-Tobon & Hendrixson, 2014; Titz *et al.*, 2006). Previously it was shown that deletion of *fliW* in *C. jejuni* 81-176 resulted in a phenotype consisting of severely attenuated motility with the presence of a probable flagellar hook structure but without flagellin filaments (Barrero-Tobon & Hendrixson, 2014). Scanning EM analysis revealed that the majority of the *flaB* (CJM1_1294) mutant clones D and E also lacked flagellar filaments and instead possessed hook-like structures, but with very few bacteria displaying a possible filament structure (Fig. 4d, e). In contrast, the majority of the bacteria imaged for the *flaB* (CJM1_1294) coupled WTs (Fig. 4f, g) and *flaB* (CJM1_1294) mutant clones A, B and C harboured a flagellar filament at one or both poles (Fig. 4a–c). Notably, the *flaB* (CJM1_1294) mutant clones D and E differed at two variant positions (Fig. 3), which suggests that they did not originate from a single transformation event. Further, the intermediate motile *flaB* (CJM1_1294) mutant clone C possessed a unique deletion of an A at position 69 410 in CJM1_0051 (*flgD*), encoding a flagellar hook assembly protein. This deletion was predicted to result in a severely truncated *flgD* ORF, encoding a protein of 55 aa instead of 294 aa. In *C. jejuni* 81-176 a *flgD* transposon mutant was previously reported to be non-motile (Hendrixson *et al.*, 2001).

The genome sequencing data obtained in this study for the M1 WTs were used to generate a reference genome sequence (designated CJM1cam) for the *C. jejuni* M1 strain used in our laboratory. The CJM1cam genome sequence and annotation are available via GenBank accession no. CP012149; for more information see Methods.

### Second-site mutations in *fliW* responsible for motility defects in *flaB* mutants

**Fig. 5.**
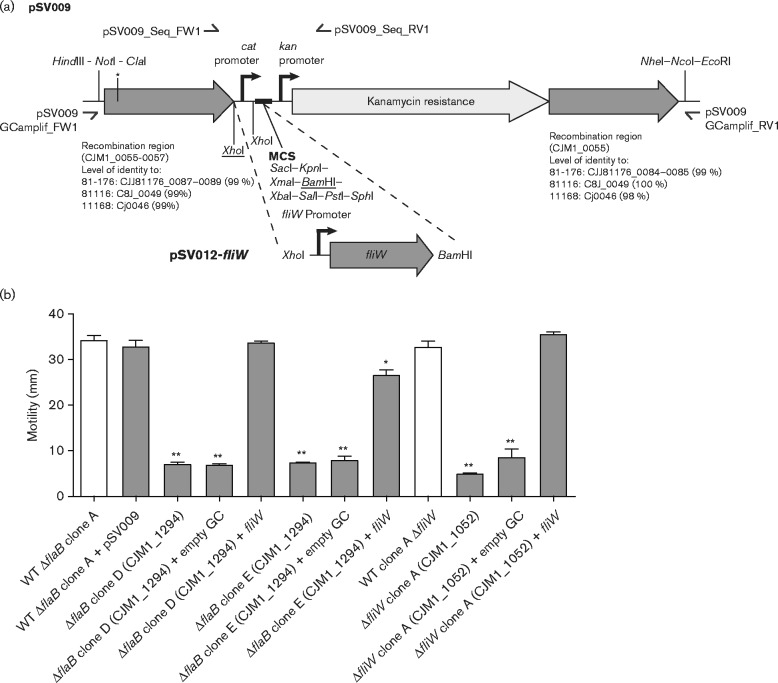
Genetic complementation of *fliW* in severely attenuated motility *flaB* (CJM1_1294) mutant clones and a *fliW* (CJM1_1052) mutant restores motility. (a) Overview of the genetic complementation strategy using plasmid pSV009. The *fliW* gene including its upstream sequence was cloned into the *Bam*HI site located in the multiple cloning site (MCS) and *Xho*I site of pSV009, yielding pSV012-*fliW*. This replaced the pSV009 *cat* promoter and enabled *fliW* expression from its native promoter. The genetic complementation region is flanked by regions for homologous recombination to facilitate insertion into the CJM1_0055–0057 pseudogene region, which is predicted to be compatible with other commonly used *C. jejuni* isolates (e.g. 11168, 81116 and 81-176). For insertion of *fliW* (CJM1_1052) into the pseudogene region, the complementation region was amplified by PCR using the pSV009_GCamplif primers and subsequently introduced by electroporation. (b) As expected, insertion of *fliW* (CJM1_1052) in the CJM1_0055–0057 pseudogene region in a *fliW* (CJM1_1052) mutant restored motility. Insertion of *fliW* (CJM1_1052) in the pseudogene region also restored motility in the severely attenuated motility *flaB* (CJM1_1294) mutant clones D and E, whereas insertion of the empty genetic complementation (‘empty GC’) region did not restore motility. The white bars indicate the coupled WT strains for *flaB* (CJM1_1294) and *fliW* (CJM1_1052) gene deletion mutants. Differential motility of mutant clones was tested against coupled WT isolates using a Mann–Whitney test (*n* = 3). Data shown are the mean and sem, with *P < 0.05 and **P < 0.01.

We hypothesized that the predicted C-terminal truncation of the FliW protein by 3 aa may be responsible for the observed attenuated motility of *flaB* (CJM1_1294) mutant clones D and E. To confirm that *fliW* is also required for motility in *C. jejuni* M1, a *fliW* gene deletion mutant was generated, which showed abolished motility (Fig. 5b). To exclude that this was a clone-specific phenotype, we assayed two other *fliW* gene deletion mutant clones; both clones were found to be non-motile (Fig. S1). Genome sequencing of *fliW* mutant clone A and its coupled WT showed that 14 SNPs and INDELs were shared between them, five variants were unique to the WT and two were only found in the *fliW* mutant clone A (Table S3). Unique variants to the *fliW* mutant clone A were an intergenic A insertion between CJM1cam_0120 (putative metalloprotease) and CJM1cam_0121 (hypothetical protein) and a synonymous SNP (A to G) in CJM1cam_0791 (hypothetical protein), indicating that the non-motile phenotype is not caused by second-site mutations. Electron micrographs of the *C. jejuni* M1 *fliW* mutant were similar to those reported previously of a *C. jejuni* 81-176 *fliW* mutant, i.e. lack of flagellar filaments and presence of a hook-like structure (Barrero-Tobon & Hendrixson, 2014) (Fig. 4h). The motility of the *fliW* mutant was slightly less than that of the *flaB* (CJM1_1294) mutant clones D and E, although this difference was not statistically significant. It is possible that the predicted 3 aa truncated FliW protein in *flaB* (CJM1_1294) mutant clones D and E could still possess limited functional activity.

To confirm that the TA-deletion in *fliW* was responsible for the severely attenuated motility of *flaB* (CJM1_1294) mutant clones D and E, the WT *fliW* gene including its native promoter was inserted into the CJM1_0055–0057 pseudogene region. For this, the new genetic complementation plasmid pSV009 was developed, which enables: (1) flexible cloning options through a whole range of restriction sites, (2) the possibility to drive the expression from the *cat* promoter or replace this with the native promoter of the gene of interest and (3) pseudogene region insertion in well-studied *C. jejuni* strains, e.g. M1, 81-176, 81116 and 11168, via homologous recombination regions with >98 % identity (Fig. 5a). Using the newly developed complementation system, insertion of *fliW* into the CJM1_0055–0057 pseudogene region in the *fliW* mutant as well as in *flaB* (CJM1_1294) mutant clones D and E was shown to restore motility to WT levels, suggesting that the flagella are restored, although this was not confirmed by EM. Insertion of the empty genetic complementation backbone did not restore motility (Fig. 5b). These results confirm that the TA-deletion in *fliW* was responsible for the attenuated motility phenotype of *flaB* (CJM1_1294) mutant clones D and E.

### Concluding remarks

Many studies aimed at investigating the role of genes in *C. jejuni* make use of targeted deletion of genes by replacing them with an antibiotic resistance cassette. Here we have demonstrated that it is of critical importance to assess the phenotype of multiple mutant clones and/or genetically complement the gene deletion. Ideally, genome sequencing may be used to screen for second-site mutations that may interfere with the pathways/mechanism under study, especially when mutant clones show inconsistent behaviour or when genetic complementation fails. As shown in this study, genome sequencing can be used as a troubleshooting tool and may also yield novel genes associated with the phenotypes under study.

## Supplementary Data

1378 Supplementary Data
