# Supplementary material for: Motility defects in Campylobacter jejuni defined gene deletion mutants caused by second-site mutations
Source: Microbiology (Reading). 2015 Dec;161(Pt 12):2316–27. doi: 10.1099/mic.0.000184 (PMC4811654; doi:10.1099/mic.0.000184)
Supplement: Supplementary file 1 — Supplementary Data [file 000184.pdf]

Table S1. Sequence of oligonucleotides used in this study

Primer Name	Target [a]	Sequence (5′ - 3′) [b]
L_WITS_1_cam_FW1	Cm^r cassette	AAGCTTGGCGTGGGAGTG
R_WITS_cam_RV1	Cm^r cassette	GAATTCCGCCCTTTAGTTCC
CC069	Cm^r cassette control primer	ATATGTGCAGGGCGTATTGC
*flaB*_L1-FW1	*flaB* (CJM1_1294) left flank	AGCAGATATTAACCGCTTGATGG
*flaB*_L2-RV1	*flaB* (CJM1_1294) left flank	CACTCCCACGCCAAGCTTACCGCAT TGATAGCTGAAACC
*flaB*_R1-FW1	*flaB* (CJM1_1294) right flank	GGAACTAAAGGGCGGAATTCAAATAT AACATTCTTGCACAATCAGGC
*flaB*_R2-RV1	*flaB* (CJM1_1294) right flank	TTCTTGCATGAAATTTTAGGACCT
*flaB*_A-RV1	*flaB* (CJM1_1294) control primer	ACAAGCTTGCCGTTTTCATC
*pflA*_L1-FW1	*pflA* (CJM1_1501) left flank	TGAGAGTTTAAATGGGCTTTTGG
*pflA*_L2-RV1	*pflA* (CJM1_1501) left flank	CACTCCCACGCCAAGCTTGCTTGATT ATTTTCGCGACCC
*pflA*_R1-FW1	*pflA* (CJM1_1501) right flank	GGAACTAAAGGGCGGAATTCAATCA AAAGTGCTTCAAATTGGC
*pflA*_R2-RV1	*pflA* (CJM1_1501) right flank	AGAGTAGGTTCTGATATGAATGCT
*pflA*_A-RV1	*pflA* (CJM1_1501) control primer	AGCTCCAACATAAGGCAAACTC
*flaD*_L1-FW1	*flaD* (CJM1_0851) left flank	AATCAAGATTTATCCGATCCAAATGT
*flaD*_L2-RV1	*flaD* (CJM1_0851) left flank	CACTCCCACGCCAAGCTTAAGCACTT TGACCGCCCATA
*flaD*_R1-FW1	*flaD* (CJM1_0851) right flank	GGAACTAAAGGGCGGAATTCAGCTA GTACAACAATTTCCCAAC
*flaD*_R2-RV1	*flaD* (CJM1_0851) right flank	ATTCCACTCCCTGTATGCCC
*flaD*_A-RV1	*flaD* (CJM1_0851) control primer	GCTTTCAGTTCCAGCTCCAG
*flaB**_L1-FW1	*flaB* (CJM1_1295) left flank	CAGAAACTGCTATAACAAATCTAGA
*flaB**_L2-RV1	*flaB* (CJM1_1295) left flank	CTCACTCCCACGCCAAGCTTCCGAT GTTGGTGTTTATCCT
*flaB**_A-RV1	*flaB* (CJM1_1295) control primer	GTTTGGTTTGAACTTGCGCC
*flaA*_L1-FW2	*flaA* (CJM1_1296) left flank	TTTGCTTAGAACATGCTATAAGGTT
*flaA*_L2-RV2	*flaA* (CJM1_1296) left flank	CTCACTCCCACGCCAAGCTTTGCTTG AGATCTTAAGCTATCTGCT
*flaA*_R1-RV1	*flaA* (CJM1_1296) right flank	GGAACTAAAGGGCGGAATTCCTCAA GCAAATTCTAGTCAGCAA
*flaA*_R2-RV1	*flaA* (CJM1_1296) right flank	TTGCTGCTTGTGAACGCAAA
*flaA*_A-RV1	*flaA* (CJM1_1296) control primer	TCCTGAACCTTGTGCACTCA
fliW-L1-FW1	*fliW* (CJM1_1052) left flank	CAGCATTGCGTGAATTTCCAA
fliW-L2-RV1	*fliW* (CJM1_1052) left flank	CTCACTCCCACGCCAAGCTTGCATTT AACAGCTAGGGTCATG
fliW-R1-FW1	*fliW* (CJM1_1052) right flank	GGAACTAAAGGGCGGAATTCACTAG ATACAGTTAATTATCCCGACT
*fliW*_R2-RV1	*fliW* (CJM1_1052) right flank	ATGGTGTGGTTGAAGCCTTG
*fliW*_A-RV1	*fliW* (CJM1_1052) control primer	TCAAGATTAATGACTACTGGAGCT
*fliW*_GC.*Xho*I_FW1	*fliW* genetic complementation	CAGCTCGAGACTTCACCTTGATTTAT ATTCTC

*fliW*_GC.*Bam*HI_RV1	*fliW* genetic complementation	CTGCCTAGGGATCCATTTGCAAGTATATAGAGC
pSV009_GCamplif_FW1	genetic complementation region	TAATAGAAATTTCCCCAAGTCCCA
pSV009_GCamplif_RV1	genetic complementation region	CTATTGCCATAGTAGCTCTTAGTGG
pSV009_seq_FW1	sequencing complemented gene	GGAGACATTCCTTCCGTATCT
pSV009_seq_RV1	sequencing complemented gene	AGCGAGACAAAAACACTGAGC

[a] Abbreviations for antibiotics: Cm^r, chloramphenicol resistance. [b] restriction sites are underlined.

Table S2 Overview of variants identified in CJM1cam compared to the M1 reference genome

Position [a]	Gene or intergenic	Gene	Description (*C. jejuni* M1 annotation)	Reference	Variant	Variant type	Feature Type	Variant effect	Predicted effect (protein level) [b]	Method of identification
60704	CJM1_0043	-	putative drug resistance transporter	A	C	SNP	transcript	stop lost, splice region	Ter351Gly; termination codon at position 351 changed to a glycine with the extension not containing a new stop codon	Illumina sequencing
130527	CJM1_0121	-	conserved hypothetical protein	T	G	SNP	coding	mis-sense	Cys418Gly	Illumina sequencing
243119	CJM1_0246	*zupT*	ZIP Zinc transporter family protein	-	A	Insertion	coding	frameshift	Asn144fs	Illumina sequencing
254704	CJM1_0259	*clpX*	ATP-dependent Clp protease, ATP-binding subunit ClpX	-	A	Insertion	coding	frameshift	Ile406fs	Illumina sequencing
328734	CJM1_0339	*lspA*	signal peptidase II	-	T	Insertion	coding	frameshift	Gly117fs	Illumina sequencing
345149	CJM1_0355	-	ATPase associated with various cellular activities family protein	-	A	Insertion	coding	frameshift	Val436fs	Illumina sequencing
538730	CJM1_0561	-	DNA polymerase III, delta subunit	T	-	Deletion	coding	frameshift	Leu172fs	Illumina sequencing
566518	CJM1_0588, CJM1_0589	*algI/-*	Alginate O-acetyltransferase AlgI/hypothetical protein	A	-	Deletion	coding	frameshift, frameshift	Leu5fs/Ala206fs	Illumina sequencing
620355	CJM1_0644	-	potassium-transporting ATPase A subunit	-	T	Insertion	coding	frameshift	Tyr249fs	Illumina sequencing
624999	CJM1_0648	*kdpD*	universal stress family protein	-	A	Insertion	coding	frameshift	Ser345fs	Illumina sequencing
635642	CJM1_0659	-	conserved hypothetical protein	C	-	Deletion	coding	frameshift	Gly134fs	Illumina sequencing
648786	CJM1_0674	-	conserved hypothetical protein	T	-	Deletion	coding	frameshift	Asn194fs	Illumina sequencing
684129	CJM1_0713	-	putative membrane protein	C	G	SNP	coding	mis-sense	Pro24Ala	Illumina sequencing
752411	CJM1_0777	-	mviN-like family protein	-	A	Insertion	coding	frameshift	Glu77fs	Illumina sequencing
790633	CJM1_0815	-	putative soluble lytic murein transglycosylase	A	G	SNP	coding	mis-sense	Ile291Thr	Illumina sequencing
813743	CJM1_0839	*astA*	arylsulfotransferase family protein	A	-	Deletion	coding	frameshift	Arg427fs	Illumina sequencing
813749	CJM1_0839	*astA*	arylsulfotransferase family protein	T	G	SNP	coding	mis-sense	Leu428Val	Illumina sequencing
817253	CJM1_0845, CJM1_0846	-, -	hypothetical protein, hypothetical protein	A	C	SNP	intergenic	-	-	Illumina sequencing
861927	CJM1_0891	*apt*	adenine phosphoribosyltransferase	C	T	SNP	coding	mis-sense	Leu46Phe	Illumina sequencing
878548	CJM1_0907	-	putative cju26	A	G	SNP	coding	mis-sense	Leu5Pro	Illumina sequencing
921927	CJM1_0954	-	M42 glutamyl aminopeptidase family protein	-	A	Insertion	coding	frameshift	Arg170fs	Illumina sequencing
939896	CJM1_0974	*rpoD*	RNA polymerase sigma factor RpoD	A	T	SNP	coding	mis-sense	Asp106Val	Illumina sequencing
961924	CJM1_0995	-	putative porin domain protein	-	T	Insertion	coding	frameshift	Asn44fs	Illumina sequencing
965941	CJM1_1000, CJM1_1001	-, *gyrA*	Putative lipoprotein, DNA gyrase subunit A	-	T	Insertion	coding	-	-	Illumina sequencing
972139	CJM1_1005	*cmeD*	outer membrane efflux family protein	A	G	SNP	coding	synonymous	Leu93Leu	Illumina sequencing
1002619	CJM1_1032, CJM1_1033	-, *murC*	integral membrane protein, UDP-N-acetylmuramate--L-alanine ligase	-	A	Insertion	Intergenic	-	-	Illumina sequencing
1103635	CJM1_1139	-	rho termination factor, N-terminal domain protein	-	A	Insertion	coding	frameshift	Ala63fs	Illumina sequencing
1130876	CJM1_1170	*mnmG*	putative tRNA uridine 5-carboxymethylaminomethyl modification enzyme MnmG	A	G	SNP	coding	mis-sense	Val438Ala	Illumina sequencing
1252081	CJM1_1289	*pseA*	putative flagellin modification protein, PseA	-	A	Insertion	coding	frameshift	Ter330fs	Illumina sequencing
1252105	CJM1_1289	*pseA*	putative flagellin modification protein, PseA	-	G	Insertion	coding	frameshift	Leu322fs	Illumina sequencing
1261071	CJM1_1295	-	flagellin B domain protein	-	T	Insertion	coding	frameshift, splice region	-	Sanger DNA sequencing
1261073	CJM1_1295	-	flagellin B domain protein	-	T	Insertion	coding	frameshift	Ter222fs	Sanger DNA sequencing
1261080	CJM1_1295	-	flagellin B domain protein	-	T	Insertion	coding	frameshift	Arg220fs	Sanger DNA sequencing
1271162	CJM1_1304, CJM1_1305	*cdsA/cdsA*	putative phosphatidate cytidylyltransferase/cytidylyltransferase family protein	-	C	Insertion	coding	frameshift, frameshift	Met9fs/Val117fs	Illumina sequencing
1300762	CJM1_1333, CJM1_1334	-, -	Iron-sulfur cluster-binding domain protein/membrane protein	T	G	SNP	coding	mis-sense, synonymous	Leu11Arg/Thr118Thr	Illumina sequencing
1300765	CJM1_1333, CJM1_1334	-, -	Iron-sulfur cluster-binding domain protein/membrane protein	T	-	Deletion	coding	frameshift, frameshift	Phe120fs/Leu13fs	Illumina sequencing
1341270	CJM1_1376	-	putative nucleotidyl-sugar dehydratase	-	T	Insertion	coding	frameshift	Phe290fs	Illumina sequencing
1514519	CJM1_1546, CJM1_1547	-, *pgpA*	sulfate adenylyltransferase, putative, phosphatidylglycerophosphatase A	-	T	Insertion	intergenic	-	-	Illumina sequencing
1544219	CJM1_1585	-	PDZ domain family protein	A	G	SNP	coding	mis-sense	Arg136Gly	Illumina sequencing
1577749	CJM1_1627, CJM1_1628	*-/bioB*	Putative biotin synthase	T	-	Deletion	coding	frameshift, frameshift	Lys2fs/Asn238fs	Illumina sequencing
1613795	CJM1_1679	*flgE-2*	putative flagellar hook protein FlgE	T	A	SNP	transcript	stop lost, splice region	Ter691Leu; termination codon at position 691 changed to a leucine with the extension not containing a new stop codon	Illumina sequencing

[a] position in *C. jejuni* M1 reference genome as published in Friis *et al.*, 2010 PloS one 5, e1225

[b] Based on Human Genome Variation Society (HGVS) notation (http://www.hgvs.org/mutnomen/); fs = frame shift

Table S3 Overview of variants identified in M1 wild-type Δ*fliW* (CJM1cam_1052) and Δ*fliW* (CJM1cam_1052) clone A

Position [a]	Gene or intergenic region	Gene	Description (*C. jejuni* M1cam annotation)	Reference	Variant	Variant type	Feature Type	Variant effect	Predicted effect (protein level)[b]	Note
***C. jejuni* M1 wild-type Δ*fliW* (CJM1cam_1052)**										
78447	CJM1cam_0061	*fliY_2*	Flagellar motor switch protein FliY, partial	-	T	Insertion	coding	frameshift	Lys138fs	Unique to WT
87749	CJM1cam_0071, CJM1cam_0072	CJM1cam_0071, CJM1cam_0072	Glutathione synthase/Ribosomal protein S6 modification enzyme (glutaminyl transferase), non-heme iron protein, hemerythrin family	-	T	Insertion	intergenic	-	-	Also present in *fliW* mutant clone A
236030	CJM1cam_0238	CJM1cam_0238	Exonuclease III	-	T	Insertion	coding	frameshift	Lys45fs	Also present in *fliW* mutant clone A
275077	CJM1cam_0280	*modB*	ABC-type sulfate/molybdate transport system, permease component	A	T	SNP	coding	missense	Leu197His	Also present in *fliW* mutant clone A
279769	CJM1cam_0286	*bioA_1*	Adenosylmethionine-8-amino-7-oxononanoate aminotransferase	-	A	Insertion	coding	frameshift	Ile138fs	Also present in *fliW* mutant clone A
730937	CJM1cam_0753, CJM1cam_0754	*uvrD, peb2*	DNA helicase II, Major antigenic peptide Peb2	-	A	Insertion	intergenic	-	-	Also present in *fliW* mutant clone A
744771	CJM1cam_0769, CJM1cam_0770	*flgS*, CJM1cam_0770	Two-component system sensor histidine kinase FlgS, Hypothetical protein CJM1cam_0770	C	T	SNP	intergenic	-	-	Also present in *fliW* mutant clone A
744795	CJM1cam_0769, CJM1cam_0770	*flgS*, CJM1cam_0770	Two-component system sensor histidine kinase FlgS, Hypothetical protein CJM1cam_0770	-	TT	Insertion	intergenic	-	-	Also present in *fliW* mutant clone A
801201	CJM1cam_0826, CJM1cam_0827	CJM1cam_0826, *folD*	Hypothetical protein CJM1cam_0826, Bifunctional protein FolD	-	A	Insertion	intergenic	-	-	Unique to WT
904409	CJM1cam_0934	*mnmE*	tRNA modification GTPase MnmE	A	T	SNP	coding	missense	Leu212Ile	Also present in *fliW* mutant clone A
917987	CJM1cam_0948	CJM1cam_0948	Non-heme iron protein, hemerythrin family	C	T	SNP	coding	synonymous	Tyr55Tyr	Also present in *fliW* mutant clone A
918173	CJM1cam_0948	CJM1cam_0948	Non-heme iron protein, hemerythrin family	C	T	SNP	coding	synonymous	His117His	Also present in *fliW* mutant clone A
976923	CJM1cam_1008	CJM1cam_1008	DnaJ like protein, partial	-	T	Insertion	coding	frameshift, splice region	-	Unique to WT
977445	CJM1cam_1009	CJM1cam_1009	Hypothetical protein CJM1cam_1009	-	G	Insertion	coding	frameshift	Leu95fs	Also present in *fliW* mutant clone A
1087523	CJM1cam_1123	CJM1cam_1123	Hypothetical protein CJM1cam_1123	-	T	Insertion	coding	frameshift	Lys383fs	Unique to WT
1263720	CJM1cam_1296, CJM1cam_1297	*flaA* and *maf-5*	Flagellin A, Motility accessory factor Maf-5	-	T, TT	Insertion	intergenic	-	-	TT variant also present in *fliW* mutant clone A
1445565	CJM1cam_1477	*galU*	UTP-glucose-1-phosphate uridylyltransferase GalU	G	A	SNP	coding	missense	Leu85Phe	Unique to WT
1449230	CJM1cam_1480	*cysW*	ABC-type tungstate transport system, periplasmic component	A	G	SNP	coding	missense	Val155Ala	Also present in *fliW* mutant clone A
1459202	CJM1cam_1491	CJM1cam_1491	Putative ATPase	A	T,G	SNP	transcript, transcript	stop gained, missense	Lysine 8 to a stop codon, Lys8Glu	G variant also present in *fliW* mutant clone A
1540983	CJM1cam_1579	*dnaG*	DNA primase DnaG	-	A	Insertion	coding	frameshift	Val482fs	
**C. jejuni M1 Δ*fliW* (CJM1cam_1052) clone A**										
87749	CJM1cam_0071, CJM1cam_0072	CJM1cam_0071, CJM1cam_0072	Glutathione synthase/Ribosomal protein S6 modification enzyme (glutaminyl transferase), non-heme iron protein, hemerythrin family	-	T	Insertion	intergenic	-	-	Also present in coupled WT
129257	CJM1cam_0120, CJM1cam_0121	CJM1cam_0120, CJM1cam_0121	Putative metalloprotease, hypothetical protein CJM1cam_0121	-	A	Insertion	intergenic	-	-	Unique to *fliW* mutant clone A
236030	CJM1cam_0238	CJM1cam_0238	Exonuclease III	-	T	Insertion	coding	frameshift	Lys45fs	Also present in coupled WT
275077	CJM1cam_0280	*modB*	ABC-type sulfate/molybdate transport system, permease component	A	T	SNP	coding	missense	Leu197His	Also present in coupled WT
279769	CJM1cam_0286	*bioA_1*	Adenosylmethionine-8-amino-7-oxononanoate aminotransferase	-	A	Insertion	coding	frameshift	Ile138fs	Also present in coupled WT
730937	CJM1cam_0753, CJM1cam_0754	*uvrD, peb2*	DNA helicase II, Major antigenic peptide Peb2	-	A	Insertion	intergenic	-	-	Also present in coupled WT
744771	CJM1cam_0769, CJM1cam_0770	*flgS*, CJM1cam_0770	Two-component system sensor histidine kinase FlgS, Hypothetical protein CJM1cam_0770	C	T	SNP	intergenic	-	-	Also present in coupled WT
744795	CJM1cam_0769, CJM1cam_0770	*flgS*, CJM1cam_0770	Two-component system sensor histidine kinase FlgS, Hypothetical protein CJM1cam_0770	-	TT	Insertion	intergenic	-	-	Also present in coupled WT
766440	CJM1cam_0791	CJM1cam_0791	Hypothetical protein CJM1cam_0791	G	A	SNP	coding	synonymous	Lys252Lys	Unique to *fliW* mutant clone A
904409	CJM1cam_0934	*mnmE*	tRNA modification GTPase MnmE	A	T	SNP	coding	missense	Leu212Ile	Also present in coupled WT
917987	CJM1cam_0948	CJM1cam_0948	Non-heme iron protein, hemerythrin family	C	T	SNP	coding	synonymous	Tyr55Tyr	Also present in coupled WT
918173	CJM1cam_0948	CJM1cam_0948	Non-heme iron protein, hemerythrin family	C	T	SNP	coding	synonymous	His117His	Also present in coupled WT
977445	CJM1cam_1009	CJM1cam_1009	Hypothetical protein CJM1cam_1009	-	G	Insertion	coding	frameshift	Leu95fs	Also present in coupled WT
1263720	CJM1cam_1296, CJM1cam_1297	*flaA, maf-5*	Flagellin A, Motility accessory factor Maf-5	-	T	Insertion	intergenic	-	-	TT variant also present in coupled WT
1449230	CJM1cam_1480	*cysW*	ABC-type tungstate transport system, periplasmic component	A	G	SNP	coding	missense	Val155Ala	Also present in coupled WT
1459202	CJM1cam_1491	CJM1cam_1491	Putative ATPase	A	G	SNP	coding	missense	Lys8Glu	G variant also present in coupled WT

[a] position in *C. jejuni CJM1cam* reference genome (CP012149) submitted as part of this manuscript
[b] Based on Human Genome Variation Society (HGVS) notation (http://www.hgvs.org/mutnomen/); fs = frame shift

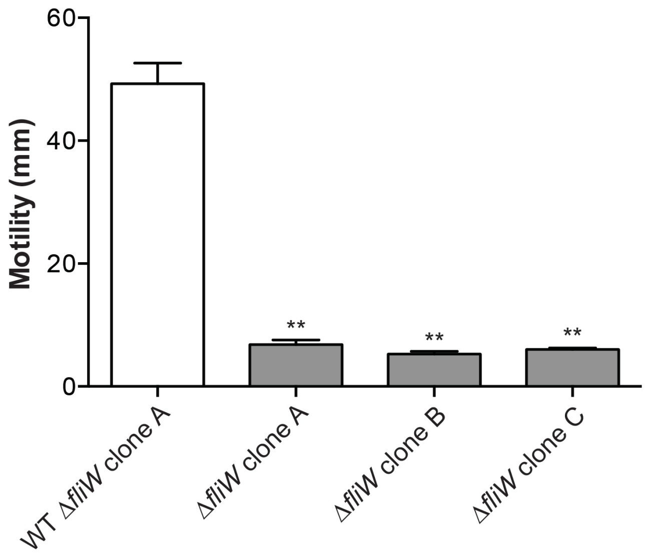

Fig. S1. Motility of *C. jejuni* M1 *fliW* defined gene deletion mutants. Motility of three *fliW* (CJM1_1052) deletion mutant clones (*n* = 4) along with their coupled wild-type strain (*n* = 2) was assayed on BHI plates with 0.4% (w/v) agar after overnight incubation. All three *fliW* (CJM1_1052) mutant clones were non-motile. Differential motility of *fliW* mutant clones was tested against the coupled wild-type (WT) strain with a Mann-Whitney test. Data shown are the mean and SEM, with ** $P < 0.01$.
